# Perosomus elumbis in Danish Holstein cattle

**DOI:** 10.1186/s12917-014-0227-2

**Published:** 2014-09-26

**Authors:** Jørgen S Agerholm, Wendy Holm, Mette Schmidt, Poul Hyttel, Merete Fredholm, Fintan J McEvoy

**Affiliations:** Department of Large Animal Sciences, Faculty of Health and Medical Sciences, University of Copenhagen, Dyrlægevej 68, DK-1870 Frederiksberg C, Denmark; Landbrugets Veterinære Konsulenttjeneste, Fynsvej 8, DK-9500 Hobro, Denmark; Department of Veterinary Clinical and Animal Sciences, Faculty of Health and Medical Sciences, University of Copenhagen, Dyrlægevej 16, DK-1870 Frederiksberg C, Denmark

**Keywords:** Cattle, Congenital, Dystocia, Genetic, Malformation, Syndrome

## Abstract

**Background:**

Perosomus elumbis (PE) is a congenital defect that has been observed sporadically in Holstein cattle for many years. However, several cases have been reported in recent years and this may indicate an unrecognised spread of a mutant allele in the Holstein population worldwide. Two cases in Danish Holstein calves are reported to provide details on the phenotype.

**Case presentation:**

Two full-term Holstein calves were born after assisted delivery due to dystocia with breech presentation. External morphological examination indicated that the lumbar, sacral and coccygeal vertebrae were absent and the abdominal region was just present as a floppy sac covered by skin and enclosing the abdominal organs. The hind limbs were hypoplastic with bilateral symmetric arthrogryposis and muscular atrophy. Radiographs, computed tomography scan and necropsy confirmed these findings. The caudal part of the thoracic spinal cord showed myelodysplasia. A range of abdominal organ malformations were found at necropsy. Inbreeding was not found during genealogical examination, but remote shared ancestors were present in the pedigrees.

**Conclusion:**

The addition of these further cases of PE to the in recent years reported record of cases should draw more attention to this defect in the Holstein breed. PE may be an emerging genetic defect in the Holstein population worldwide and cases should be sampled to enable genetic mapping of the gene possibly underlying the disease. PE cases seem to be associated with a high risk of dystocia due to increased rate of breech presentation.

**Electronic supplementary material:**

The online version of this article (doi:10.1186/s12917-014-0227-2) contains supplementary material, which is available to authorized users.

## Background

Perosomus elumbis (PE) is a lethal congenital defect characterized by absence of the caudal parts of the spine and spinal cord, i.e. lumbar, sacral and coccygeal spinal segments with associated musculoskeletal malformations of the hind quarters. PE was first reported in the veterinary literature in 1832 as mentioned by Jones [1], who reviewed the literature on bovine PE. PE has also been reported in other animal species such as sheep [2], pigs [3,4], dogs [5] and horses [6].

Scientific publications on PE in cattle have only consisted of case reports mainly focusing on the morphology [1,7-15] although a recent publication suggested that fetal infection with bovine virus diarrhea virus (BVDV) may contribute to the development of PE [15]. Also, an ovine PE case following feeding of pregnant sheep with *Veratrum californicum* on gestation days 16 and 17 was mentioned by Dennis et al. [2]. However, it remains purely speculative that teratogens should cause PE.

Investigations into a possible genetic etiology of PE in cattle have not been reported, so a possible genetic etiology remains non-elucidated. A number of case reports published during recent years have shown that PE occurs in the Holstein breed worldwide [9,10,12-16]. Also, Jones [1] refer that 25 Holstein cases were recorded in the period from 1986–1992 at the Kansas State University, USA and that a genetic etiology was suspected. Although such reporting patterns may indicate an increased number of cases, it is still inconclusive as publication of cases is influenced by many factors. However, it is worth noticing that equivalent patterns of publication have been observed for recently discovered inherited defects in Holstein cattle such as complex vertebral malformation (CVM) syndrome [17] and brachyspina syndrome [18]. Research into the etiology of PE in cattle is therefore needed.

Here we report two additional cases in Holstein cattle submitted for examination as part of the Danish Bovine Genetic Disease Programme [19] to characterize this phenotype in detail and to draw the attention towards this possible emerging defect in the Holstein breed.

## Case presentations

### Case DK 1

A stillborn male Holstein calf (body weight 27.3 kg) was delivered on gestation day 283 by cesarean section due to dystocia with breech presentation. The calf originated from a herd free of BVDV infection and a number of other infections officially eradicated from Denmark [20].

**Figure 1:**
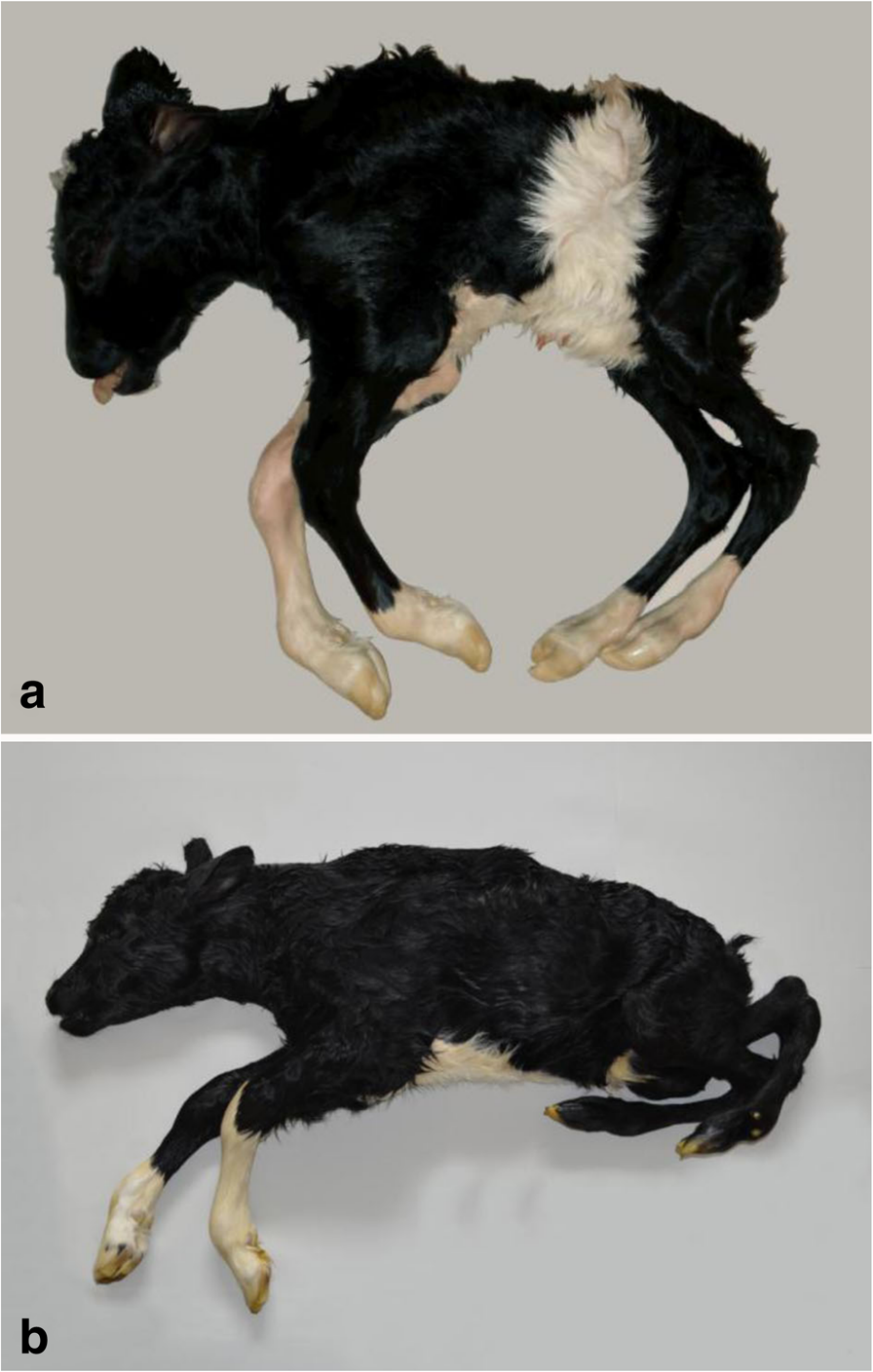
**Perosomus elumbis in Holstein calves.** Body parts caudal to the thoracic spine are dysplastic due to absence of lumbar, sacral and coccygeal vertebrae. The posterior limbs have bilateral symmetric flexion of the knee and hock joints and extension of the pastern joints with extensive muscular atrophy. Notice that the severity of hind limb malformation varies between cases. **a** and **b** represent cases DK1 and DK2, respectively.

The calf had severe dysplasia of the body parts caudal to the thoracic spine, while the cranial half of the body was normally developed (Figure 1a). The lumbar, sacral and coccygeal vertebrae were absent and the abdominal region was present as a floppy sac covered by skin and enclosing the abdominal organs. The pelvis was developed but was malformed and asymmetrical. The hind limbs were hypoplastic and had bilateral symmetric flexion of the stifle and tibiotarsal joints and extension of the metatarsophalangeal joints with ankylosis.

**Figure 2:**
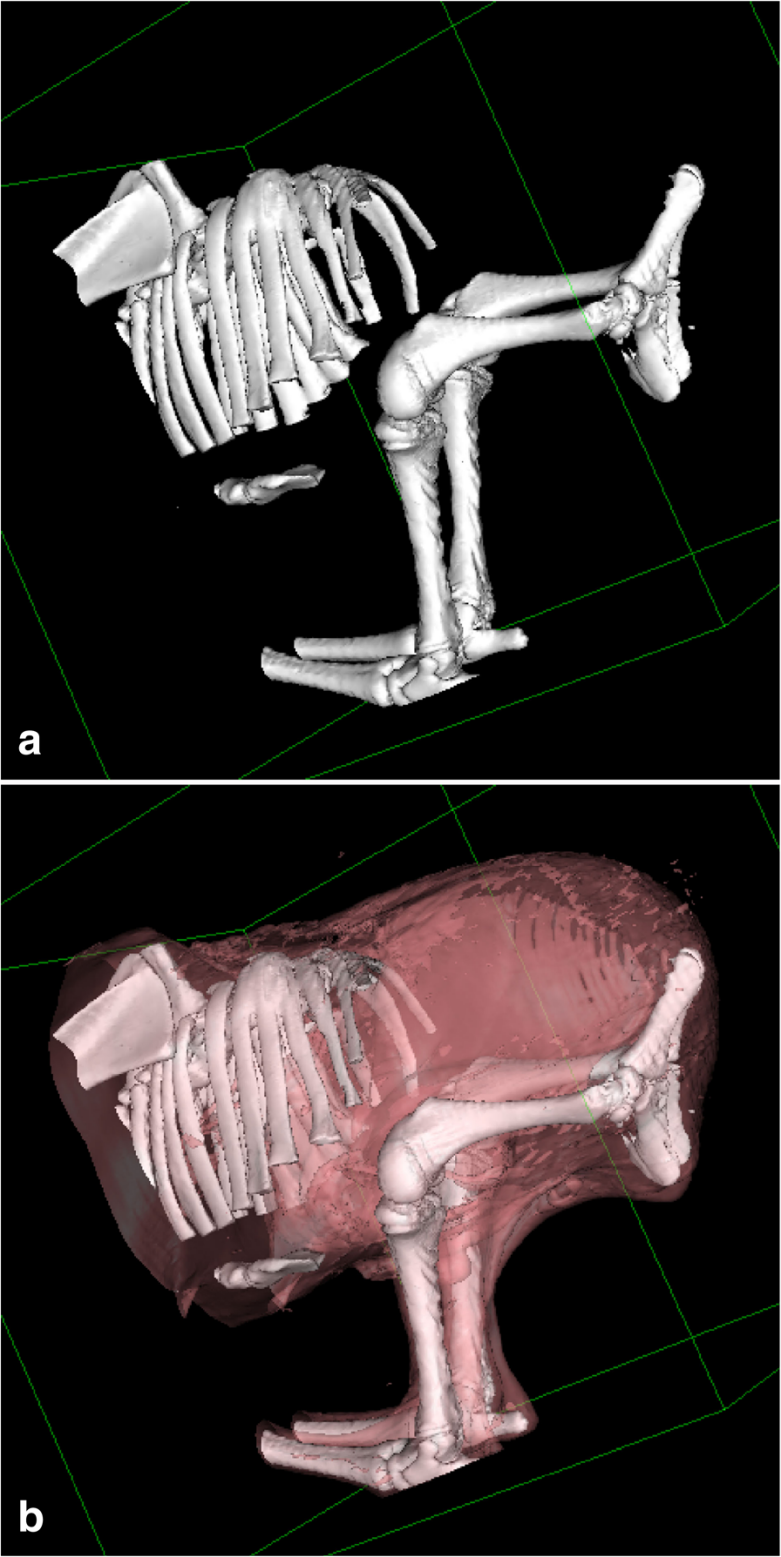
**Surface rendered computed tomography images of perosomus elumbis case DK1. a)** Surface rendering^a^ to show bone surfaces only. Absence of lumbar, sacral and coccygeal spine can be appreciated. The most posterior thoracic vertebrae are abnormally shaped and the posterior rib pairs are abnormally crowded. **b)** Same data set as in a), but rendered with bone and soft tissue surfaces. The latter has been set to have a degree of transparency in the reconstruction thus allowing visualisation of the calf’s overall morphology and its relation to the underlying skeletal abnormalities.

The calf initially underwent computed tomography (CT) scanning and radiology that included the entire skeleton and allowed an appreciation of the complicated malformations present. The mid thoracic vertebrae appeared normal with regular shaped vertebral bodies, facet joints and neural arches. At the caudal extremity of the thoracic spine, vertebrae became incomplete. First the dorsal spinous processes and neural arches disappeared so that the four most caudal rib pairs were bunched together in a disorganized fashion, in places articulating with the vertebral bodies. A single isolated vertebral body without a neural arch or spinous process was seen as the most caudal extremity of the spine. Mineralised spinal tissues were not seen caudal to this as the gap between the caudal extremity and the pelvis was bridged by soft tissues without trace of lumbar or sacral vertebrae. The iliac wings were closely apposed. Surface rendered constructions of the caudal parts of the body are shown in Figures 2a, b and a CT scan movie showing the caudal part of the calf in a 360° view is presented in Additional file 1.

**Figure 3:**
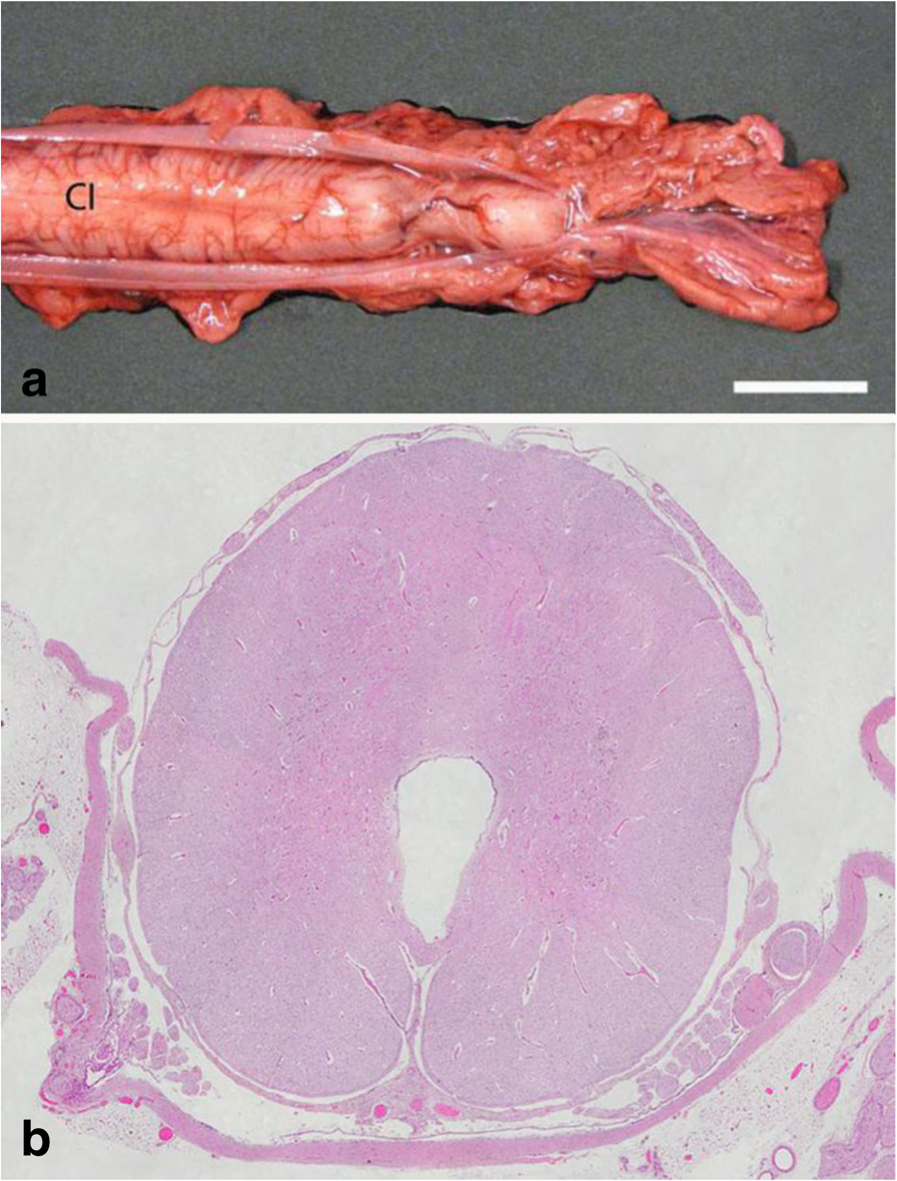
**Myelodysplasia in perosomus elumbis case DK1. a)** The spinal cord terminates posterior to the cervical intumescence (CI) as an hourglass-like structure that continues into filaments of nerves embedded in fat. The dura mater has been opened in the dorsal midline allowing exposure of the spinal cord. Bar = 2 cm. **b)** Histological section showing a distended central canal that protrudes ventrally and interrupts the grey matter. Haematoxylin and eosin, 4 μm.

Necropsy basically confirmed the findings made by the external examination and CT scanning. The cervical and thoracic spinal segments were developed but the thoracic spine showed an increasing degree of malformation towards the caudal pole and truncated as a tapering hook-like structure due to a 180° scoliosis. The height of thoracic spinous processes was increased and formed a hump-like structure. The vertebral canal and the spinal cord were normally developed until the last segments of the cervical intumescence after which the vertebral canal decreased in size and finally disappeared. The size of the spinal cord decreased simultaneously and terminated around 3.5 cm caudal to the cervical intumescence as an hourglass-like structure that continued into filaments embedded in fat (Figure 3a). The caudal ribs had an uneven non-parallel course and the thorax was narrowed.

The lungs had diffuse congenital atelectasis with areas of compression atrophy. The heart showed dilation of the right ventricle. The intestine had ano-rectal agenesia with moderate distension of the descending colon due to meconium accumulation. The left kidney, adrenal gland, testicle and umbilical artery were absent and the scrotum was hypoplastic. The right kidney was located in the caudal abdomen. The aorta and caudal vena cava had a winding course through the abdomen and the left testicle was located adjacent to the abdominal aorta.

Specimens of brain, spinal cord, heart, lung, liver, spleen, adrenal gland, thymus, testis, hind limb musculature and placenta were sampled, fixed in 10% neutral buffered formalin, processed for histology by routine methods, sectioned at 4 μm and stained with hematoxylin and eosin. The spinal cord was additionally stained with Luxol fast blue. The cervical intumescence and spinal cord segments caudal to this showed myelodysplasia with tissue disorganization and hydromyelia (Figure 3b). The hind part musculature showed extensive lipomatous atrophy.

Relationship between the calf and the parents was ensured by genotyping their DNA using the microsatellites BM2113, BM1824, BM1818, ETH10, ETH225, ETH3, INRA012, INRA005, and INRA63 [21]. Pedigree examination was done by construction of a complete seven-generation pedigree based on data from the Danish Cattle Data Base. The pedigree revealed no close inbreeding, but the parents shared ancestors in generation 7 and more remote generations. These consisted of US sires that had been used widely in the Holstein breed.

The dam of case DK 1 was acquired from the owner in an attempt to produce diseased embryos by superovulation and breeding with the same sire. The cow was treated with an intramuscular injection of 0.75 mg cloprostenol (Estrumat vet® 0.25 mg/ml, MSD Animal Health, Ballerup, Denmark) on day 11 after heat. The cow was then treated twice a day with a combination of follicle-stimulating hormone and luteinising hormone (Pluset®, Minitüb, Tiefenbach, Germany) on days 11–14 after the cloprostenol induced heat and additionally with 0.75 mg cloprostenol on day 14. Insemination was done three times during the heat (22 h). The study was performed in accordance with the Danish animal welfare regulations.

The cow was transported to the University of Copenhagen on gestation day 26 and euthanised at arrival by intravenous injection of an overdose of pentobarbital sodium. The uterus was removed from the carcass and opened. Embryos were not present despite multiple corpora lutea in the ovaries. However, locally diffuse fibrotic peritonitis due to the previous cesarean section enclosed the ovaries and probably hindered proper ovulation and fertilisation.

### Case DK 2

A male Holstein calf (body weight 33.6 kg) was born on gestation day 283 after assisted calving due to dystocia with breech presentation. The calf was euthanized immediately after calving by intravenous injection of an overdose of pentobarbital sodium, submitted to the university and subsequently examined as described for case DK1. The herd was free of BVDV infection and other notifiable diseases [20].

The calf had agenesia of lumbar, sacral and coccygeal vertebrae with accompanying dysplasia of the caudal part of the body and severe bilateral symmetrical hind limb arthrogryposis (Figure 1b). The pelvis was malformed and the tail present as a 2 cm long soft tissue structure.

**Figure 4:**
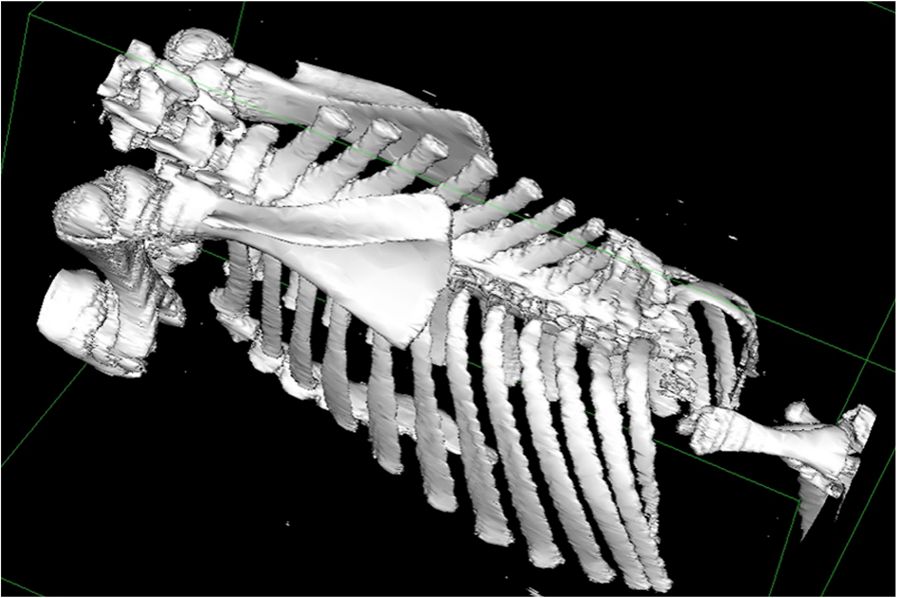
**Surface rendered computed tomography image of perosomus elumbis case DK2.** Surface rendering^a^ allowing visualisation of the skeletal abnormalities. The caudal thoracic vertebrae are malformed and the lumbar vertebrae are seen as a disorganised bony structure protruding ventrally into the abdomen. The caudal ribs have an uneven course with fusion of the proximal part in the right side.

CT scanning and radiology revealed normally developed cervical and thoracic vertebrae until T4 while the subsequent thoracic vertebrae were malformed. The 12th thoracic vertebra had a deformed dorsal spinous process and the vertebral canal was filled with cancellous bone. Structures resembling vertebrae were not present caudal to this, but a disorganised bony structure protruded into the cranial abdomen. The most caudal ribs had an uneven course with fusion of the proximal parts in the right side (Figure 4). The pelvis was malformed with the iliac wings being in direct contact with each other. A CT scan movie showing the skeletal malformations in a 360° view is presented in Additional file 2.

**Figure 5:**
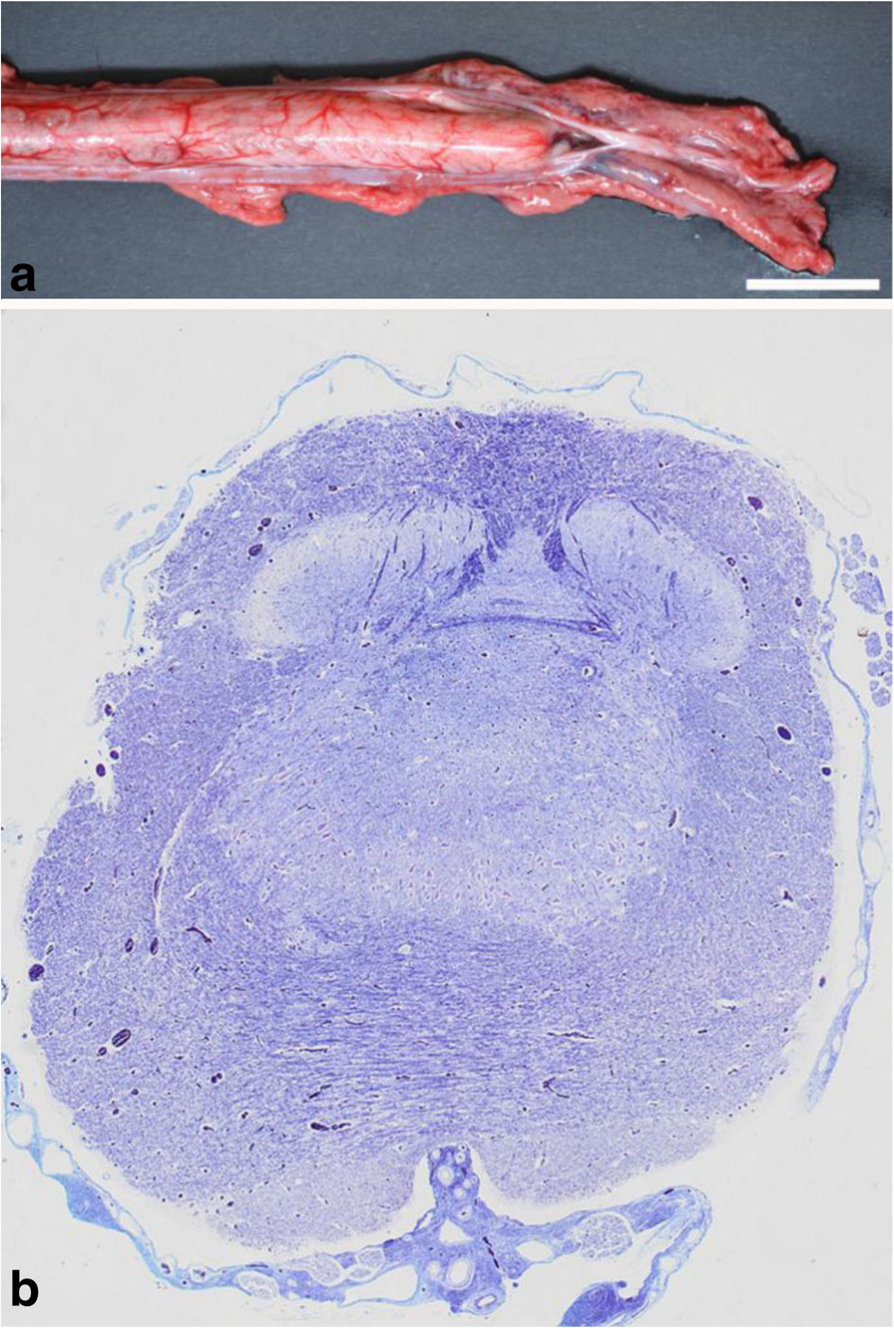
**Myelodysplasia in perosomus elumbis case DK2. a)** The thoracic spinal cord ends abruptly and continues caudally for a few cm as broad nerve bundles. The dura mater has been opened in the dorsal midline allowing exposure of the spinal cord. Bar = 2 cm. **b)** Histological section of the most caudal segments of the thoracic spinal cord showing absence of a central canal and disorganized white and grey matter. Luxol fast blue, 4 μm.

Necropsy revealed normally developed cervical and cranial thoracic spinal segments, while the caudal thoracic spine was malformed and lumbar vertebrae were only present as a 7 cm bony structure protruding into the abdomen in a ventral direction. The caudal thoracic spine had a slight scoliosis. The number of left and right ribs was 12 and 13, respectively. The spinal cord was normally developed until the caudal part of the thoracic spine. Here it suddenly decreased in size, and continued as nerve bundles (Figure 5a). The vertebral canal ended simultaneously. One enlarged kidney was located at the cranial border of the pelvic cavity with two testes of uneven size present at the cranial pole (length left testis: 3.0 cm, right 1.5 cm). Two adrenal glands were present of which the right was attached to the diaphragm. The scrotum was hypoplastic.

Histology of the cervical and cranial thoracic spinal cord showed a few distended axons and macrophages in the ventral white matter. The central canal gradually increased in size and became irregular with interrupted ependymal lining in the caudal segments of the thoracic spinal cord with final loss of the central canal and simultaneous disorganizing of grey and white matter (Figure 5b). Severe lipomatous atrophy was present in the hind part musculature.

Genotyping of the calf and its parents by microsatellites [21] rejected the registered paternity, so examination for inbreeding was not possible. Maternal pedigree examination showed that the dam shared ancestors in generation 7 and more remotely with some of the sires found as shared ancestors of the parents of case DK 1.

## Conclusions

The morphology of the present cases is consistent with PE and basically similar to other cases reported in Holstein cattle [1,7,9,10,12-16] even though there is a certain morphological variation among cases as also recognized for other bovine congenital spinal syndromes [18,22]. Although the spinal lesions are the most striking and the cause of hind limb dysplasia, visceral defects were present as well. Visceral defects may occur due to disturbed development of the embryonic back. Similar lesions have been found in other bovine syndromes with widespread disturbed segmentation of the embryonic back such as the brachyspina syndrome [22], but such abnormalities seem also to be rather frequent in cases with spinal lesions restricted to the coccygeal vertebrae, i.e. the caudo-recto-urogenital syndrome [23].

It is at present unknown if PE is inherited and our attempt to investigate this using the dam of case DK1 was unfortunately unsuccessful. A similar experiment using the dam of case 2 is planned. The pedigree examination failed to identify a close common ancestor for the parents of case DK 1. Remote shared ancestors were found in generation 7 or earlier and these were also shared with the dam of case DK2, but interpretation of such findings in relation to transmission of a mutant allele is uncertain. Holstein calves often have one or more individuals shared by their parents in remote generations due to the intensive use of certain breeding lines. Some sires will be present in the pedigree of both parents simply because of their extensive use as previously observed for the CVM syndrome [17]. The lack of a closely related common ancestor does not rule out a genetic cause of PE as a mutant allele may be present in the general population. It is important to remember that PE was reported already in 1832 and that multiple cases were encountered in the Holstein breed from 1986 to 1992 [1], so dissemination of a mutant allele has had time to occur. Also, presence of more than one mutant allele in the population may explain why cases can occur without the parents having a closely related ancestor.

The low number of reported cases may indicate that PE is a rare disorder in Holsteins and therefore of minor economic significance to the breed. However, experience with other congenital spinal syndromes in Holstein cattle during recent years have illustrated that full-term defective calves may simply just be the “tip of the iceberg” and that a significant number of cases are lost undiagnosed during pregnancy [24,25]. If this turns out to be the case also for PE, this syndrome may have an unrecognized high gene frequency in the Holstein population worldwide as previously recognized for the CVM and brachyspina syndromes and thus possibly represent an emerging genetic disease.

PE cases are often reported to have caused dystocia requiring cesarean section and the syndrome is therefore associated with maternal welfare concerns and economic losses beyond those of the lost offspring. The fetal presentation is usually not reported, but Williams [7] mentions that five out of seven cases were in caudal presentation as in the present cases. As caudal presentation is usually present in only 5% of bovine deliveries [26], caudal presentation is apparently much more common in PE affected calves. Due to the hind limb arthrogryposis, calves will thus be presented in breech position, which is a life threatening condition for the dam as such calvings may remain unnoticed thus leading to fetal death, emphysema and maternal intoxication. A majority of bovine fetuses are in caudal presentation during gestation months 4 – 6½ after with most of them (~95%) reposition to cranial presentation [26]. The underlying mechanisms for these fetal movements seem to be disturbed in PE affected fetuses.

## Consent

Consent was obtained from the owner of animals for publication of this case report.

## Endnote

^a^OsiriX: An open-source software for navigating in multidimensional DICOM images.

## Additional files

Additional file 1
**Surface rendered computed tomography scanning movie of bovine perosomus elumbis case DK1.**
Additional file 2
**Surface rendered computed tomography scanning movie of bovine perosomus elumbis case DK2.**
